# Technical Performance Reduces during the Extra-Time Period of Professional Soccer Match-Play

**DOI:** 10.1371/journal.pone.0110995

**Published:** 2014-10-24

**Authors:** Liam D. Harper, Daniel J. West, Emma Stevenson, Mark Russell

**Affiliations:** Health and Life Sciences, Northumbria University, Newcastle-upon-Tyne, United Kingdom; Norwegian University of Science and Technology, Norway

## Abstract

Despite the importance of extra-time in determining progression in specific soccer tournament matches, few studies have profiled the demands of 120-minutes of soccer match-play. With a specific focus on the extra-time period, and using a within-match approach, we examined the influence of prolonged durations of professional soccer match-play on markers of technical (i.e., skilled) performance. In 18 matches involving professional European teams played between 2010 and 2014, this retrospective study quantified the technical actions observed during eight 15-minute epochs (E1: 00∶00–14∶59 min, E2: 15∶00–29∶59 min, E3: 30∶00–44∶59 min, E4: 45∶00–59∶59 min, E5: 60∶00–74∶59 min, E6: 75∶00–89∶59 min, E7: 90∶00–104∶59 min, E8: 105∶00–119∶59 min). Analysis of players who completed the demands of the full 120 min of match-play revealed that the cumulative number of successful passes observed during E8 (61±23) was lower than E1–4 (E1: 88±23, P = 0.001; E2: 77±21, P = 0.005; E3: 79±18, P = 0.001; E4: 80±21, P = 0.001) and E7 (73±20, P = 0.002). Similarly, the total number of passes made in E8 (71±25) was reduced when compared to E1 (102±22, P = 0.001), E3 (91±19, P = 0.002), E4 (93±22, P≤0.0005) and E7 (84±20, P = 0.001). The cumulative number of successful dribbles reduced in E8 (9±4) when compared to E1 (14±4, P = 0.001) and E3 (12±4, P≤0.0005) and the total time the ball was in play was less in E8 (504±61 s) compared to E1 (598±70 s, P≤0.0005). These results demonstrate that match-specific factors reduced particular indices of technical performance in the second half of extra-time. Interventions that seek to maintain skilled performance throughout extra-time warrant further investigation.

## Introduction

Soccer is a high-intensity intermittent sport which is normally played over 90 min. However, when scores are tied at the end of certain matches in soccer tournaments (e.g., FIFA World Cup, UEFA Champions League, FA Cup etc.), an extra-time period, consisting of two additional 15-min periods of play, follows the end of normal time. Notably, between 1986 and 2010, 33 matches have required that extra-time be played in FIFA World Cup competitions. In the 2014 FIFA World Cup, of the matches played in the knockout stages, 50% required extra-time to be played. Although the physiological and performance responses to the normal duration (i.e., 90 min) of soccer match-play have been extensively researched [1], [2], [3], [4], [5], comparable data during the extra-time period is lacking. This is somewhat surprising considering the role that this additional period of play has in determining success in tournament situations.

The ability to maintain skill proficiency during soccer match-play is considered an important factor in overall player performance and match success [6]. However, soccer-specific exercise appears to influence skilled performances executed during normal durations (i.e., 90 min) of match-play. For example, using a within-subject study design, Russell et al., [7] observed reductions in total possessions and the number of ball distributions in the second versus the first half of English Championship matches. Further analysis across 15-min intervals revealed that the total number of ball possessions and distributions reduced in the final 15 min when compared with the opening phase of play. Although self-pacing strategies [8] and tactical modifications [9] have been suggested to explain such changes, these data support studies where the accumulated effects of match-related fatigue have been proposed to explain decrements in short passing performance during Italian Serie A matches [4] and the increased number of goals conceded in the final 15 min of match-play [10].

Interestingly, the decrement in skill performance observed throughout soccer-specific exercise appears to differ according to the skill being performed. For example, Rampinini et al. [4] identified that three quarters of the technical measures examined were similar between halves in Serie A players who experienced physical fatigue decrements. Russell et al. [7] also identified no changes in five of the seven indices of technical performance (i.e., number of touches taken per possession, number of challenges, percentage of challenges won, length of forward distributions and percentage success of distributions) examined throughout match-play. Therefore, it appears that the technical response to the normal duration of soccer match-play is not uniform. However, it is plausible that the prolonged duration of games that enters in to the extra-time period elicit different technical responses compared to those observed during 90 min. No data is currently available that has profiled the skill response associated with soccer matches where the extra-time period has been played.

Therefore, using performance analysis techniques, the aim of this study was to examine the influence of prolonged durations of actual soccer match-play (i.e., those matches which required extra-time to be played) on markers of technical performance. We hypothesised that extra-time would influence technical performance; specifically, by reducing certain indices of performance.

## Methods

### Participants

Eighteen matches that required an extra-time period to be played were included in the analyses. Only outfield players that completed the full 120 min of the match (i.e., 90 min plus 30 min extra-time) were analysed. Using a within-match approach, the technical actions elicited during matches involving professional European clubs (ranging from the third tier of their domestic league to top tier and International teams) were analysed (15±1 players per match). Written informed consent was obtained from the professional soccer clubs who supplied footage to be analysed for the purpose of the study. The study was approved by the Faculty of Health and Life Sciences Ethics Committee of Northumbria University in Newcastle upon Tyne, UK.

### Match Analysis Procedures

In agreement with the experimental design used by previous authors [7], skill-related performances were analysed retrospectively during competitive matches played since 2010 using existing footage obtained from television recordings (8 matches) and footage supplied following correspondence with specific clubs (10 matches). To minimise variation, each match was manually coded (Sportstec Gamebreaker, Sportstec, New South Wales, Australia) by one experienced performance analyst according to operational definitions detailed by Rampinini et al. [4] and Opta Sports Data (http://www.optasports.com/news-area/blog-optas-event-definitions.aspx). A total of 17 technical variables (Table 1) were analysed for each match.

**Table 1:** Operational definitions of the technical variables analysed (derived from Rampinini, et al. [4] and Opta Sports Data).

Variable		Operational definition
Passing	Successful passes	A pass that is performed with the foot or head that is received successfully by a teammate
	Unsuccessful passes	A pass that is performed with the foot or head that is not received successfully by a teammate (i.e., is instead either intercepted by an opposition player or leaves the field of play)
	Total passes	Sum of successful and unsuccessful passes
	Pass accuracy (%)	Successful passes divided by total passes, multiplied by 100
Dribbling	Successful dribbles	A situation when a player takes control of the ball and is able to keep possession of the ball before performing another action such as a pass or shot
	Unsuccessfuldribbles	A situation when a player takes control of the ball and is subsequently dispossessed by an opposing player or dribbles the ball out of play
	Total dribbles	Sum of successful and unsuccessful dribbles
	Dribbleaccuracy (%)	Successful dribbles divided by total dribbles, multiplied by 100
Shooting	Shots on target	Any goal attempt that:a) Goes into the netb) Would have gone into the net but for being stopped by a goalkeeper's savec) Would have gone into the net but for being stopped by a defender who is the last man.
	Shots off target	Any goal attempt where the ball is going wide of the target, misses the goal or hits the woodwork
	Total shots	Sum of shots attempted
	Shot accuracy (%)	Shots on target divided by total shots, multiplied by 100
Crossing	Successfulcrosses	A long pass using the foot that is performed by a player within the last 35 m of the pitch and which is directed into the penalty area and is received by a teammate
	Unsuccessfulcrosses	A long pass using the foot that is performed by a player within the last 35 m of the pitch and which is directed into the penalty area and is not received by a teammate but is instead controlled or cleared by an opposition player or goes out of play for a goal-kick or throw in
	Total crosses	Sum of successful and unsuccessful crosses
	Crossaccuracy (%)	Successful crosses divided by total crosses, multiplied by 100
Time-in-play		The amount of time in seconds per 15 min epoch that the ball is within the field of play. A ball is deemed out of the field of play in the cases of a(n): goal kick, free kick, throw in, penalty, corner, goal celebration, substitution, extraordinary circumstances (e.g., pitch invasions). In these occurrences, the clock is restarted when the ball re-enters the field of play or in the case of goal celebrations, when the subsequent kick-off is taken by the team that conceded the goal.

In order to investigate the transient effects of 120 min of soccer match-play on technical performance, all matches were divided into eight 15 min epochs (i.e., E1: 00∶00–14∶59 min, E2: 15∶00–29∶59 min, E3: 30∶00–44∶59 min, E4: 45∶00–59∶59 min, E5: 60∶00–74∶59 min, E6: 75∶00–89∶59 min, E7: 90∶00–104∶59 min, E8: 105∶00–119∶59 min). To ensure that the duration of each epoch was standardised, data collected in injury time was not included in the analyses. The intra-observer reliability of measurements was assessed by repeated coding of all variables on two occasions for selected epochs in six randomly chosen matches (Table 2). To minimise learning effects, coding during the retest was performed in a blind fashion and 4 weeks separated the test and retest measurements.

**Table 2:** Test-retest reliability data (intraclass correlation coefficients) for the technical variables examined.

Variable	E1	E6	E8
Successful passes	0.993*	0.999*	0.998*
Unsuccessful passes	0.961*	0.982*	0.997*
Total passes	0.969*	0.998*	0.998*
Pass accuracy (%)	0.982*	0.950*	0.938*
Successful dribbles	0.916*	0.954*	0.999*
Unsuccessful dribbles	0.927*	0.885*	0.827*
Total dribbles	0.892*	0.977*	0.982*
Dribble accuracy (%)	0.929*	0.648	0.715
Shots on target	1.000*	0.615	0.432*
Shots off target	0.851*	0.956*	0.956*
Total Shots	0.937*	1.000*	0.906*
Shot accuracy (%)	0.964*	1.000*	0.135
Successful crosses	0.962*	1.000*	0.968*
Unsuccessful crosses	0.852*	0.982*	1.000*
Total crosses	0.692*	0.977*	0.993*
Cross accuracy (%)	0.946*	0.999*	0.938*
Ball time in play	0.999*	0.968*	0.978*

Where E1 = 00∶00–14∶59 min, E6 = 75∶00–89∶59 min, E8 = 105∶00–119∶59 min.

*represents significance at *P*≤0.05 level.

### Statistical Analysis

The appropriateness of statistical methods to analyse data yielded from multiple matches has been debated [11], [12]. To minimise the influence of dependence in the data sets, given that each match has distinct characteristics, we have used a within-match design; therefore, the data presented (means ± SD) reflects the cumulative totals of technical actions performed within each match per epoch. Due to violations of normality, differences between time-points were investigated using Friedman’s repeated-measures analysis of variance on ranks tests. Where appropriate, post-hoc analyses were applied using Bonferroni-corrected Wilcoxon’s signed rank tests. Statistical significance was set at *P*≤0.05. Effect sizes (ES) for statistical differences were determined according to Field [13] with values of 0.2, 0.5, and >0.8 considered to reflect small, medium, and large differences respectively [14]. Test-retest reliability was assessed using intra-class coefficient (ICC) calculations. All statistical analyses were conducted using SPSS Version 21.0 (IBM, Armonk, NY, USA).

## Results

Extra-time influenced 4 of the 17 technical variables analysed (Table 3). Specifically, the number of successful passes observed during E8 was lower than E1 (−31%, *P* = 0.001, ES = 0.52), E2 (−22%, *P* = 0.005, ES = 0.42), E3 (−24%, *P* = 0.001, ES = 0.50), E4 (−25%, *P* = 0.001, ES = 0.53) and E7 (−17%, *P* = 0.002, ES = 0.48). Similarly, the total number of passes made in E8 was reduced when compared to E1 (−30%, *P* = 0.001, ES = 0.52), E3 (−21%, *P* = 0.002, ES = 0.49), E4 (−21%, *P*≤0.0005, ES = 0.54) and E7 (−15%, *P* = 0.001, ES = 0.50). Furthermore, dribbling success in E8 was reduced when compared to E1 (−32%, *P* = 0.001, ES = 0.50) and E3 (−24%, *P*≤0.0005, ES = 0.59). The time the ball was in play was less in E8 compared to E1 (−16%, *P*<0.0005, ES = 0.55). All other technical variables analysed, including shooting and crossing indices, were similar between extra-time and the rest of the match.

**Table 3:** Technical performance variables (mean ± SD) as a function of timing throughout matches.

Variable	Timing throughout match								Time effect		Post-hoc differences (*P*≤0.007) compared to E7 and E8
	E1	E2	E3	E4	E5	E6	E7	E8	ChiSquare *(X^2^_(7)_)*	*P* value	
**Passing**											
Successful passes	88±23	77±21	79±18	80±21	66±20	65±17	73±20	61±23	32.91	<0.0005	E8 vs. E1, E2, E3, E4, E7
Unsuccessfulpasses	14±5	12±5	11±4	13±4	10±3	10±5	11±4	11±5	22.97	0.002	
Total passes	102±22	89±21	91±19	93±22	76±21	76±21	84±20	71±25	37.23	<0.0005	E8 vs. E1, E3, E4, E7
Pass accuracy (%)	85±7	86±7	87±5	86±4	86±6	86±5	87±6	84±7	4.10	0.769	
**Dribbling**											
Successfuldribbles	14±4	13±5	12±4	13±5	11±4	12±4	11±3	9±4	15.79	0.027	E8 vs. E1, E3
Unsuccessful dribbles	5±3	5±2	4±1	4±3	4±2	5±2	4±2	5±2	7.20	0.408	
Total dribbles	18±4	18±6	16±5	17±5	15±4	16±4	15±3	15±5	13.12	0.069	
Dribbleaccuracy (%)	72±15	74±12	76±9	72±18	72±14	71±16	72±12	64±16	8.17	0.318	
**Shooting**											
Shots on target	1±1	1±1	1±1	1±1	2±1	1±1	1±1	1±1	11.45	0.120	
Shots off target	2±1	2±1	2±2	2±2	2±1	3±2	2±1	2±1	11.57	0.116	
Total shots	3±2	2±1	3±2	4±2	4±2	4±2	3±2	3±1	12.57	0.083	
Shot accuracy (%)	37±37	37±32	30±28	39±32	44±28	22±27	36±27	31±26	7.44	0.384	
**Crossing**											
Successfulcrosses	1±1	1±1	2±1	1±1	1±1	2±1	2±1	2±1	5.44	0.606	
Unsuccessfulcrosses	3±2	4±2	3±3	5±2	4±3	4±2	4±2	4±2	11.95	0.102	
Totalcrosses	5±2	5±2	5±3	6±3	6±3	6±3	6±2	6±3	5.17	0.640	
Cross accuracy(%)	29±27	15±15	37±28	22±25	31±29	29±25	28±21	27±19	10.48	0.163	
**Time in play (s)**	598±70	554±81	554±52	553±60	493±83	502±81	551±65	504±61	27.66	<0.0005	E8 vs. E1

Where E1 = 00∶00–14∶59 min, E2 = 15∶00–29∶59 min, E3 = 30∶00–44∶59 min, E4 = 45∶00–59∶59 min, E5 = 60∶00–74∶59 min, E6 = 75∶00–89∶59 min, E7 = 90∶00–104∶59 min, E8 = 105∶00–119∶59 min.

## Discussion

The aim of this observational study was to identify the influence of the extra-time period on the technical requirements elicited during professional soccer match-play. In agreement with our hypothesis, we demonstrated using a within-match study design, that indices of technical performance, namely passing and dribbling, reduced in the last 15-min of extra-time. To our knowledge, this is the first study to report data concerning the influence of extra-time on professional soccer match-play; therefore such data can be used as a benchmark for future work. Given the importance of this additional competitive period in deciding success and progression in soccer tournament scenarios, this data is likely to be of interest to those responsible for the technical preparations of soccer players.

Passing performance (i.e., the number of successful and total passes) reduced by more than 20% during E8 when compared to the first half of match-play. However, the accuracy of passing remained unchanged during this time (Table 1). Although changes in formation appear to influence passing frequency [15], our data may also indicate that the effects of match-related fatigue observed during the extra-time period of actual match-play impairs the ability to get involved with the ball rather than a player’s passing proficiency *per se* [4]. Unfortunately, physical performance measures are unavailable to substantiate this speculation and further research is needed to provide further information regarding the extra-time responses of soccer players.

Although direct comparison of our data to that reported in other performance analysis studies is limited, our findings support, and extend, previously published data identifying decrements of both physical [4], [16] and technical [4], [7] markers in the latter stages of normal time (i.e., 90 min). In players who were deemed to be fatigued, Rampinini et al. [4] reported between-half reductions of ∼12–16% in the number of technical involvements performed per player in the Italian Serie A league. In combination with our observations, it therefore appears that the proficiency, and number, of specific technical actions reduces as a match progresses.

Match-related fatigue could plausibly explain our observations but it is important to note that the reductions in the physical performances observed in midfield players was not previously accompanied by reduced technical performance [16]. To date, the effects of 120 min of soccer-specific exercise remain to be fully elucidated using controlled and repeatable experimental procedures (e.g., Soccer Match Simulation; [17]). Notably, the lack of differences observed during E8 relative to the technical performances observed in E6 means that the transient changes observed during extra-time, at least in the variables assessed in this study, appear to be similar to those elicited in the latter stages of normal time (e.g., E6). It is therefore plausible that interventions that seek to counteract the deleterious effects of match-related fatigue during games that last 90 min, may also be efficacious during extra-time. However, this supposition is yet to be examined.

In previous studies where soccer match-play has been subdivided into smaller segments, statistical artefacts have been proposed to explain the identification of transient reductions in performance when compared to the opening phase of play [9]. A desire to enforce tactical superiority [9] and residual ergogenic effects resulting from the warm-up [7] have been cited to artificially elevate the pace of play in the initial stages of a match and thereby influence subsequent comparisons to observations made during this interval. In the absence of appropriate methods of data analysis that overcome such approaches, it is important to note that the transient changes identified in E8 in this study were rarely yielded from comparison to E1 alone. In combination with the medium effect sizes for the majority of differences observed, we propose that the transient changes identified are reflective of the demands of the extra-time period and not a reflection of statistical artefacts.

Not all of the technical actions examined demonstrated a uniform response to exercise when match-play was separated into 15-min epochs; for example, ∼23% of the variables examined exhibited transient changes. These findings support our previous data, albeit during 90 min of English Championship match-play, where only two of the seven technical measures examined (namely total number of possessions and distributions performed) reduced in the last 15 min [7]. However, contrary to previous literature [4], [16], we observed a reduction in the number of successful dribbles performed in the final 15 min of extra-time. As far as we are aware, and while acknowledging the differential effects of soccer-specific exercise on technical performance [5], no study has previously identified transient changes in dribbling performance throughout actual match-play. We attribute the identification of this finding to the longer duration of exercise examined in this study. Such data is likely to have important implications for the tactics employed by teams competing in the extra-time period.

The precise mechanisms regulating performance throughout soccer-specific exercise remain to be established and are likely to be multifaceted in nature. Notwithstanding the influence of previously mentioned factors, such as team tactics [9] and self-pacing strategies [8], it must be noted that a greater degree of variation is observed in technical performance measures [18], [19] when compared to physical performance. Furthermore, although it has previously been proposed that a lack of sensitivity exists in the gross measures derived from computerised time-motion analysis studies [16], we have again used performance analysis techniques, albeit with a within-match rather than within-player study design, and observed differences in the cumulative technical responses observed during 15 min epochs in matches involving professional soccer players. Furthermore, we have also demonstrated the test-retest reliability of such measures over different assessment periods.

When interpreting the current findings, a number of limitations should be considered. Firstly, it is prudent to note that this data represents a within-match rather than a within-player approach. The low occurrence of games requiring extra-time, the number of eligible games that footage was available for, and the number of repeated player observations, would have yielded data with extremely low statistical power if a within-player approach has been adopted. While acknowledging this limitation, we believe that we are the first group to present data relating to the technical responses observed during the extra-time period in soccer. Additionally, our findings support previously published data, especially in relation to the transient variations observed [4]. Secondly, as the ball was in play for longer in E8 than E1 (note that this was the only variable to show differences between E8 and E1 alone), one could argue that the differences observed were reflective of an interaction with this variable. However, as aforementioned, the reductions in performance observed in E8 were yielded from comparison to other epochs in addition to E1; therefore, it is unlikely that such differences reflect the time that the ball was in play. Finally, this study was a descriptive study; therefore, it was not possible to determine the cause of temporal changes in the performance of technical actions but we acknowledge the potential role of match-specific factors such as game context (e.g., score line, venue, team/opposition quality etc.) and the area of the pitch in which technical actions are performed (e.g. attacking and defensive third) [20], [21].

## Conclusions

In summary, this study presents novel findings describing temporal patterns in the technical actions observed during 120 min of professional soccer match-play. We provide evidence demonstrating that the number of successful and total passes, number of successful dribbles and the time that the ball was in play, reduced by more than 20% in matches that required extra-time to be played; particularly in the last 15-min of extra-time. Although the current study was unable to elucidate the specific reasons for these findings, coaches and conditioning staff could use this information to inform team tactics and technical training sessions. Implementation of strategies that seek to minimise such occurrences (e.g., substitutions, aerobic and anaerobic conditioning programs and nutritional supplementation protocols [22] etc.) should be considered; however, the efficacy of such strategies remains to be confirmed when 120 min of actual match-play is performed.

## Supporting Information

Table S1 **Raw passing data.**
(XLSX)

Table S2 **Raw shooting data.**
(XLSX)

Table S3 **Raw crossing data.**
(XLSX)

Table S4 **Raw dribbling data.**
(XLSX)

Table S5 **Raw time in play data.**
(XLSX)

## References

[1] Di Salvo V, Baron R, Tschan H, Calderon Montero FJ, Bachl N, et al (2007) Performance characteristics according to playing position in elite soccer. Int J Sports Med 28: 222–227. 17024626 10.1055/s-2006-924294

[2] Mohr M, Krustrup P, Bangsbo J (2003) Match performance of high-standard soccer players with special reference to development of fatigue. J Sports Sci 21: 519–528. 12848386 10.1080/0264041031000071182

[3] Rahnama N, Reilly T, Lees A, Graham-Smith P (2003) Muscle fatigue induced by exercise simulating the work rate of competitive soccer. J Sports Sci 21: 933–942. 14626373 10.1080/0264041031000140428

[4] Rampinini E, Impellizzeri FM, Castagna C, Coutts AJ, Wisloff U (2009) Technical performance during soccer matches of the Italian Serie A league: effect of fatigue and competitive level. J Sci Med Sport 12: 227–233. 18083631 10.1016/j.jsams.2007.10.002

[5] Russell M, Benton D, Kingsley M (2011) The effects of fatigue on soccer skills performed during a soccer match simulation. Int J Sports Physiol Perform 6: 221–233. 21725107 10.1123/ijspp.6.2.221

[6] Lago-Penas C, Lago-Ballesteros J, Dellal A, Gomez M (2010) Game-related statistics that discriminated winning, drawing and losing teams from the Spanish soccer league. J Sports Sci Med 9: 288–293. 24149698 PMC3761743

[7] Russell M, Rees G, Kingsley MI (2013) Technical demands of soccer match play in the english championship. J Strength Cond Res 27: 2869–2873. 23287830 10.1519/JSC.0b013e318280cc13

[8] Edwards AM, Noakes TD (2009) Dehydration: cause of fatigue or sign of pacing in elite soccer? Sports Med 39: 1–13. 19093692 10.2165/00007256-200939010-00001

[9] Weston M, Batterham AM, Castagna C, Portas MD, Barnes C, et al (2011) Reduction in physical match performance at the start of the second half in elite soccer. Int J Sports Physiol Perform 6: 174–182. 21725103 10.1123/ijspp.6.2.174

[10] Reilly T (2003) Motion analysis and physiological demands. In: Williams AM, Reilly T, editors. Science and Soccer. London: Routledge. 59–72.

[11] Wilkinson M, Akenhead R (2013) Violation of statistical assumptions in a recent publication? Int J Sports Med 34: 281. 23420193 10.1055/s-0032-1331775

[12] Aughey RJ, Varley MC (2013) Acceleration profiles in elite Australian soccer. Int J Sports Med 34: 282. 22895869 10.1055/s-0032-1316315

[13] Field A (2009) Discovering statistics using SPSS. London: Sage publications.

[14] Cohen J (1988) Statistical power analysis for the behavioral sciences. New Jersey: Lawrence Erlbaum.

[15] Bradley PS, Carling C, Archer D, Roberts J, Dodds A, et al (2011) The effect of playing formation on high-intensity running and technical profiles in English FA Premier League soccer matches. J Sports Sci 29: 821–830. 21512949 10.1080/02640414.2011.561868

[16] Carling C, Dupont G (2011) Are declines in physical performance associated with a reduction in skill-related performance during professional soccer match-play? J Sports Sci 29: 63–71. 21077004 10.1080/02640414.2010.521945

[17] Russell M, Rees G, Benton D, Kingsley M (2011) An exercise protocol that replicates soccer match-play. Int J Sports Med 32: 511–518. 21472627 10.1055/s-0031-1273742

[18] Ali A, Williams C, Hulse M, Strudwick A, Reddin J, et al (2007) Reliability and validity of two tests of soccer skill. J Sports Sci 25: 1461–1470. 17852682 10.1080/02640410601150470

[19] Russell M, Benton D, Kingsley M (2010) Reliability and construct validity of soccer skills tests that measure passing, shooting, and dribbling. J Sports Sci 28: 1399–1408. 20967673 10.1080/02640414.2010.511247

[20] Mackenzie R, Cushion C (2013) Performance analysis in football: a critical review and implications for future research. J Sports Sci 31: 639–676. 23249092 10.1080/02640414.2012.746720

[21] Taylor JB, Mellalieu SD, James N, Shearer DA (2008) The influence of match location, quality of opposition, and match status on technical performance in professional association football. J Sports Sci 26: 885–895. 18569554 10.1080/02640410701836887

[22] Russell M, Kingsley M (2014) The efficacy of acute nutritional interventions on soccer skill performance. Sports Med 44: 957–970. 24728928 10.1007/s40279-014-0184-8

